# Use of Heat-Applied Coatings to Reduce Wear on Agricultural Machinery Components

**DOI:** 10.3390/ma17122849

**Published:** 2024-06-11

**Authors:** Dawid Romek, Jarosław Selech, Dariusz Ulbrich

**Affiliations:** 1 Faculty of Civil and Transport Engineering, Poznan University of Technology, 60-965 Poznan, Poland; dawid.romek@put.poznan.pl (D.R.); dariusz.ulbrich@put.poznan.pl (D.U.); 2 Institute of Mechanical Science, Vilnius Gediminas Technical University, Plytinės Str. 25, LT-10105 Vilnius, Lithuania

**Keywords:** tribology, abrasive wear, padding, soil, pH, moisture, energy, friction

## Abstract

This article presents the effect of the conditions of abrasive compounds on the wear of samples made by different methods. The 28MnB5 steel was used, which is intended for agricultural components, to which two arc and laser coatings were applied. The study included the analysis of microstructure, microhardness, roughness, and tribological experiments on a dedicated stand. The arc coating was found to significantly improve the tribological properties compared to the samples without the coating. Varied wear results were obtained for the laser coating depending on the parameters of the abrasive compound. Studies of the surface roughness of the samples showed that the concentration and pH of the abrasives have a significant effect on the changes in the surface parameters after the tribological tests. The results of the tribological experiments indicated that wear resistance for some of the abrasive mass conditions was improved by the application of heat-applied coatings. In addition, it was found that the power consumption on the stand was the highest for abrasive mass conditions of a 10% moisture content and a pH of 10. For these test conditions, the mass loss was four times higher than for the parameter with W0% and pH7. The energy consumption of the stand was 60 kWh lower for this variant than for the parameter with W10% and pH10. The results of the study have important practical applications that can help in the selection of materials for agricultural machinery components, depending on the abrasive mass conditions.

## 1. Introduction

The influence of individual soil properties (abrasive mass) on the total mass wear has not yet been thoroughly studied. Many researchers [1,2,3,4,5] working on the subject of abrasive wear in abrasive mass identified with soil have been trying for years to accurately describe the model of the influence of individual components on the total abrasive wear. An attempt to accurately describe the factors was made by Nosal [6], who derived a formula for the total abrasive wear and made an attempt to accurately describe the factors. Only the factors affecting the total wear were proposed, without considering the dependence of these factors on the total wear.

(1) $$Z_{v}=f\left(P,E,G,t\right)$$

where:

Z_v_—the volumetric wear of the working tool;

P—the external force that acts on the tool;

E—the tool properties;

G—the soil properties;

t—the duration of the wear.

Hebda made an attempt to determine the effect of alloying elements on the wear reduction [7]. Controlling the abrasive wear resistance by modifying the chemical composition allows one to obtain the properties of steel designed for the assumed work for which the tools are made. Modifying the steel through the addition of alloying elements is one of the three basic treatments to increase the resistance of the material to abrasive wear. The study of the effect of the content of various elements and their amounts is the subject of scientific research. Wang showed that optimal gas flow rates of 0.2 L/min for carrier gas and 30 L/min for shielding gas can reduce the angle of divergence to 6° and the diameter of the powder spot to about 2 mm at a distance of 15 mm from the outlet for the powder applied to the component [8]. Titarmare et al. [9] studied a specially modified AZ31 casting alloy that was tested on a pin-on-disk tribotester to determine the abrasive wear and friction characteristics of the developed materials at different sliding distances (40, 50, and 60 mm raceway diameter) and different abrasive grains (400, 500, and 600). The results confirmed that the wear rate of the AZ31 alloy is approximately 1.3–1.5 times higher than that of the AZ31-2.0B4C compound for all of the experimental conditions. The results show that the composite reinforced with submicrometer particles provides better wear resistance under all experimental conditions. Rajendhran [10] tested the materials in conjunction with the WC and demonstrated research in the ASTM G65 [11] abrasive material on the changes in the component wear depending on the abrasive mass parameters. The grain size shows that the dominant wear micromechanisms change from the binder removal (turning) to the mixed binder and carbide extrusion (sliding) when the abrasive particle size changes from smaller (67 μm) to larger (245 μm). The hardness ratio between the abrasives and cermets (Ha/Hs) highlights that silica and alumina abrasives provide mild wear (Ha/Hs < 1.5), while SiC abrasives cause severe wear (Ha/Hs > 1.5).

The second group with a direct impact on the increase in the abrasive wear resistance is the modification of the surface layer through heat-applied coatings. The applied coatings affect the modification of the surface layer prior to curing, reducing the roughness or forcing the flow of the abrasive mass in a way that affects the overall abrasive wear. The paper by [12] demonstrated the effect of the laser coating application on the working elements of the abrasive mass working components. The authors of [13] indicated significant differences in the surface and surface stresses of the cooperating samples and their wear. Bartkowski, on the other hand, demonstrated the effect of laser-applied coatings on the reduction in the wear of machine elements for the abrasive mass [14]. The authors of [15] attempted to develop and study the addition of a low-energy powder applied to increase the hardness of the components and achieve a low energy consumption.

The third group of wear reduction includes designs that affect changes in the organic wear abrasion through design optimisation and analysis using DEM [16]. Researchers address aspects related to structural optimisation using CAD environments [16,17,18]. Structural optimisation also affects organic wear by preventing hard organic particles from directly accessing the mating parts. Structural modifications are also important as early as conceptual work on the materials used to construct friction nodes [19]. Selecting the right types of association has a positive effect on removing hard particles or minimising the abrasive wear [20,21]. When designing a friction node, it is important to give the ability to remove hard particles from the node formed during operation as a result of abrasion. A design step that is important for abrasive associations is to give an equilibrium roughness to compensate for large roughness on the surface of cooperating parts. Large differences in roughness increase the resistance needed for the process of shearing the roughness and smoothing the surface. The effect of the roughness to the force was described by Glumac [22]. It was found that, as the roughness of the surface increases, the force needed to overcome the resistance to the movement of the cooperating parts also increases. Menga et al. [23] performed an ad hoc-developed time-saving procedure with an FEM analysis of the shape evolution during the wear process by the Winkler simplifying assumption. This is an important direction of research development that allows for the simulation and shaping of the reliability and durability of machine components exposed to abrasive mass particles and particles of worn material.

The energy intensity of the elements used on a component in agricultural machinery is particularly important in terms of reducing the air pollution produced by the machinery that work on the ground [24,25]. The use of various surface treatments that decrease friction resistance has a significant impact on reducing the energy required to perform work [26,27]. The increase in the emissivity of agricultural machinery is influencing the development of research related to ways to reduce friction resistance. Particularly important for reducing frictional resistance in the initial phase, before the stage of establishing the equilibrium roughness, is the roughness created in the manufacturing process. Roughness can also be imparted by surface treatment. For various types of treatments, the temperature and depth of the operation are important [14]. The problem that occurs with this type of treatment is the thickness of the layer, which imparts an increased value to the abrasion resistance of the component working in the abrasive mass by wearing down the coating first rather than the main material.

A significant influence of the type of abrasive mass and its physicochemical parameters that affect compactness has also been noted [28]. As the moisture content increases, the compactness of the soil changes, which influences the greater resistance that must be overcome to perform agricultural work. Pentos et al. [29] studied, during field tests, the effect of the compactness on the energy required to do the work under different conditions of the grinding mass. For the parameters adopted, it was determined that the tractive force and tractive efficiency were more influenced by the type of soil (58.3 and 74.5%, respectively). Two additional parameters that significantly affected the pulling force and traction performance were the vertical load (18.3 and 10.1%, respectively) and soil moisture (19.8 and 10.3%, respectively).

Despite the realisation of research on the wear of agricultural machinery components, there is a need to improve manufacturing techniques in terms of reducing the wear of heavy and agricultural machinery components. Research results available in the literature mainly focus on the modifications of the components and their surface layer. There is a lack of knowledge about the influence of varying parameters of the abrasive mass, such as the moisture content or pH, on the wear of samples, its course, and the energy consumption parameters during the operation of the tool in the abrasive mass.

The main objective of this study was to determine the effect of abrasive mass conditions on the wear of specimens made of 28MnB5 steel, which were subjected to surface treatment by applying arc and laser coatings. In addition, the energy consumption on the test bench was verified at different moisture and pH contents of the abrasive mass. The article includes a description of the research methodology, the main results, and their discussion in relation to available results of other researchers. The final section provides a summary and directions for further research that will seek to optimise the wear of components working in the abrasive mass. The article points out important research on basic steels (hard-to-wear steels) aimed at the validity of its use under different abrasive mass conditions and the effect of heat-applied coatings to reduce the abrasive wear. The results obtained indicate an alternative use of 28MnB5 steel for wear-resistant steels such as Hardox and Raex.

## 2. Materials and Methods

The 28MnB5 steel was selected as the material. It is a wear-resistant steel intended for components used for work in agricultural machinery. This material is used to make components such as [30]:

− cultivator teeth;
− plough blades;
− the teeth of active rotary harrows;
− elements performing agrotechnical operations to a depth of up to 20 cm.

This steel is characterised by an increased boron content. In addition, it has elements in its composition such as manganese and chromium that affect the resistance to abrasive wear. This steel is a wear-resistant steel, but it is used for less-abrasive wear parts compared to Hardox or Raex steel. The steel used in the study has the chemical composition shown in Table 1.

The samples used in the laboratory tests were cuboid in shape, with dimensions of 100 × 25 × 6 mm. The samples were cut by high-energy waterjet cutting. The use of this method of sample preparation was intended to not interfere with the structure of the surface layer. The steel supplied for the sample preparation was cold-rolled as delivered.

The sample, after being prepared to a suitable shape, was polished to a roughness of Ra10. The polishing of the samples was intended to clean the residues of the cutting process and prepare the surface for better adhesion of the heat-applied coatings. It was also important to remove corrosion effects from the storage and delivery process.

Coatings applied to the 28MnB5 steel were selected on the basis of research from the literature, which also pointed out the important application of coatings in the case of components used for screws for plastic injection moulding machines, in which high-impact strength with adequate component hardness is also very important [31]. These included arc and laser coatings. Other layers such as thin-walled layers were also considered, but these layers, which were presented in Yanchuk’s study, were rejected at the concept stage due to their small size [32]. The arc coating to obtain the appropriate geometric parameters related to the shape formed on the surface of the specimen was applied by a robotic station using a FANUC R-2000iC/125L robot (Fanuc, Oshino, Japan) and a welding machine operating in MIG/MAG technology with a self-feeding welding wire. Various researchers in their work have investigated this method and the improvement of welded joints [33,34].

The choice of an arc coating was made to verify a popular technology among commercial solutions for increasing the resistance to abrasion wear. This method is used for components that work in the soil. The advantage of this method is that the process can be carried out under workshop conditions by qualified welders with the appropriate qualifications. Another surface coating used was a laser-melted coating. The coating was made by a paste melt applied to the part using a high-power laser beam. The application parameters of the coating are shown in Table 2, while the chemical composition of the coatings applied to the 28MnB5 steel are shown in Table 3.

**Figure 1:**
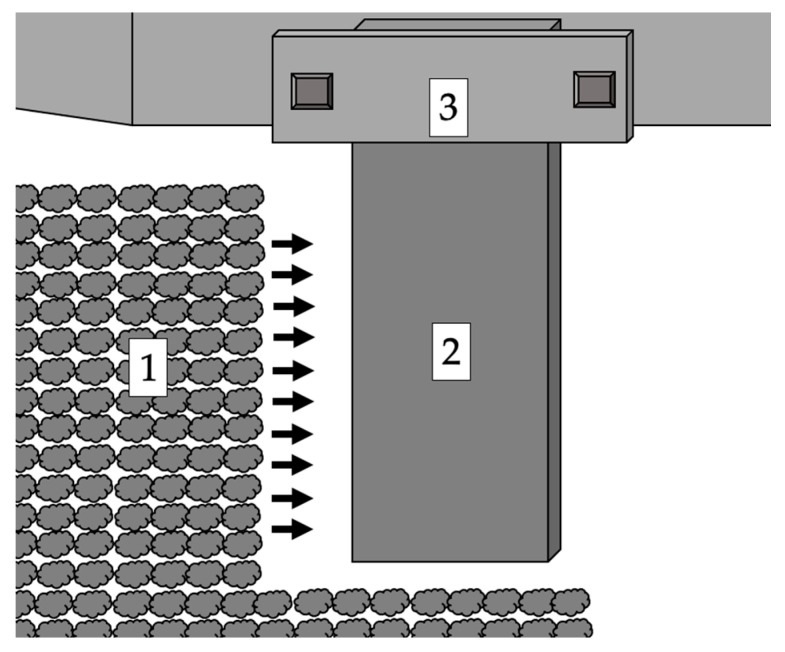
Diagram of the action of the abrasive mass on the surface of the samples. 1—direction of the abrasive mass; 2—depth of immersion of the sample; 3—location of the sample holder.

The front surface of the samples (marked 2 in Figure 1) was chosen as the application site for the coatings. The use of a face directly exposed to the abrasive flow for testing reflects the operation of active rotary harrows and the pressure of the soil on the elements in it. A schematic diagram of the location of the coatings on the samples and the area of the pressure of the abrasive medium are shown in Figure 1. For statistical purposes, 6 test samples were used for each of the abrasive mass and coating conditions. This is the value to determine the statistical sample.

**Figure 2:**
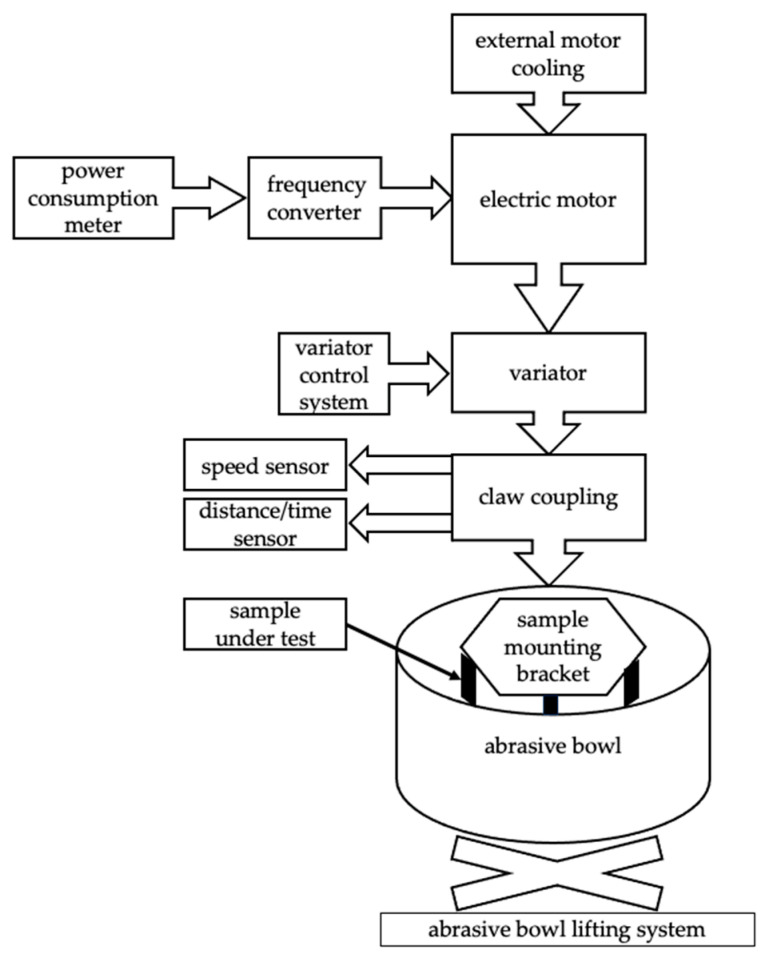
Schematic of the test stand.

**Figure 3:**
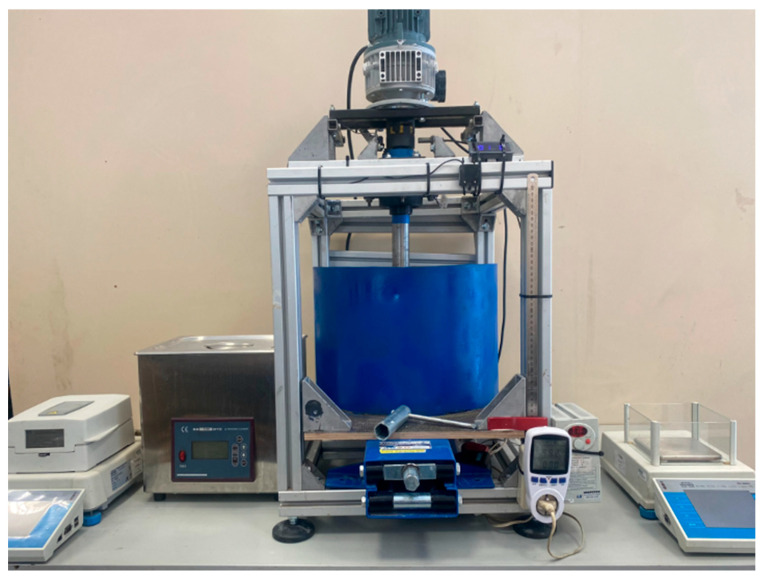
View of the test stand.

To perform the tribological tests, a prototype test stand was designed and manufactured. The testing device is designed to verify the wear of components working in an abrasive mass. A schematic diagram of the stand is presented in Figure 2. A real view of the stand used during the tribological test is presented in Figure 3.

One of the design considerations for the stand was the way to change the parameters of the abrasive medium. The main goal was to be able to quickly modify the machine parameters and the abrasive mass used during the test. The moisture content (W) and soil reaction (pH) were chosen as variable parameters of the abrasive medium. The quartz sand used for the tests was in accordance with ASTM G65 [11]. Moisture contents of 10% and 0% were used as the abrasive medium conditions. The pH values for the tests were set at 7 and 10. The test parameters are shown in Table 4, while the abrasive medium determinations are presented in Table 5.

The parameters defined (pH and moisture) for the abrasive mass reflect the actual operating conditions that occur during agrotechnical procedures performed during the field work in the fall and spring; these are the extreme parameters in which agrotechnical treatments are most often performed. The operating parameters of the test stand are shown in Table 6.

**Figure 4:**
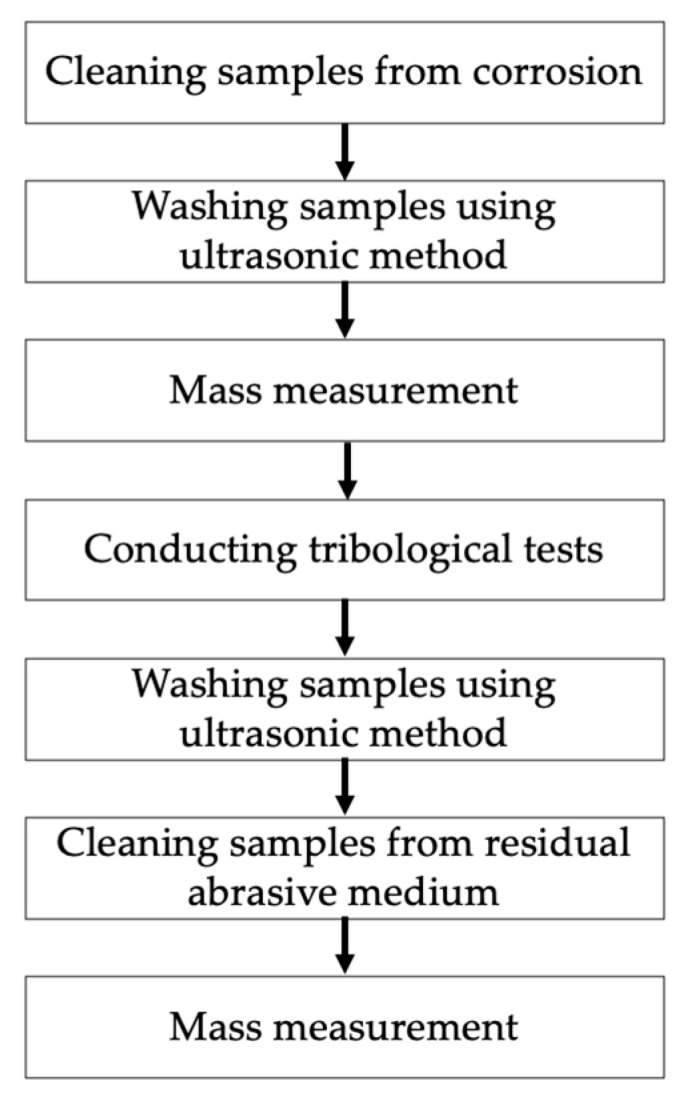
Methodology of the tribological tests.

To carry out the tribological experiment, it was necessary to plan the sequence of tasks. A diagram showing the main stages of the experiment is presented in Figure 4.

The samples were also subjected to the analysis of their hardness and microstructure. For this purpose, tests were performed on the samples in the initial state and after the coating application process. Due to the visible heat-affected zones, 4 areas were designated for testing. A NIKON ECLIPSE MA200 (Nikon, Minato, Japan) test stand operating in accordance with PN-EN ISO 6507-1 [35] was used for the tests. The Eclipse MA200 (Nikon, Minato, Japan) is a state-of-the-art inverted microscope designed for material inspection and with an innovative design. The microscope uses integrated intelligence to automatically combine captured images with relevant sample data. Microhardness tests were performed on an automatic hardness tester. The microhardness was measured in the coating (in the cross-section of the sample). Microhardness testing was performed with a Presi device to accurately characterise all visible phases formed during the coating processes and in the base material. The microhardness test was carried out in accordance with PN-EN ISO 6507-1 [35]. The test was performed for an automated path consisting of 12 measuring locations. The load with which the test took place was 0.1 N. The samples were etched with Kallings reagent.

To complement the tests, analyses of the surface roughness of the specimens were also performed at the point of abrasive medium pressure (face). The tests were carried out using a Bruker Alicon device with the designation of the Infini-teFocus G5. Key parameters related to the surface roughness, such as R_z_, R_t_, R_a_, S_z_, and Sa, were considered in the study. Both the surface geometry parameters and the roughness formed after the tribological test were examined. This was compared with the test results obtained prior to the tribological research. This allowed for the determination of the differences in values after the application of the coatings and after the experiment. The analysis made it possible to determine the magnitude of the changes occurring on the surface of the elements tested in the basic parameters of the surface roughness profile.

## 3. Results

### 3.1. Microstructure and Microhardness

**Figure 5:**
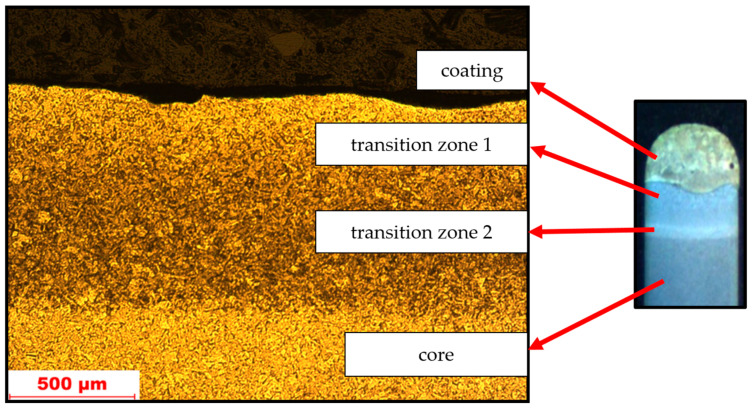
Transition zones and microstructure of the material along with the applied arc coating.

Before the tribological test, the microhardness analysis of the samples was performed. On the basis of the observations, four zones with different microhardness values were found, and the zones are shown in Figure 5.

**Figure 6:**
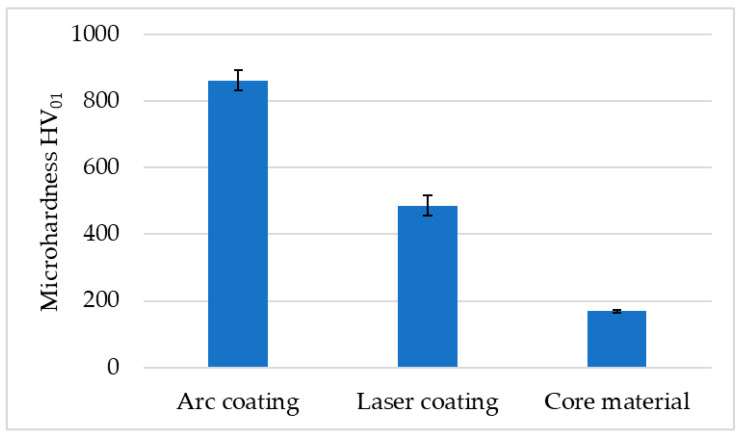
Results of the average microhardness test of the samples, depending on the place of measurement.

The test results presented in Figure 6 were obtained for the arc coating, the laser coating, and the core of the material, which had hardness in the initial state. Analysing the results obtained, it can be concluded that the highest hardness was measured for the steel together with the arc coating, which was about 780 HV_01_. The hardness of the laser coating was about 440 HV_01_, while the value measured for the steel in the initial state was about 180 HV_01_.

**Figure 7:**
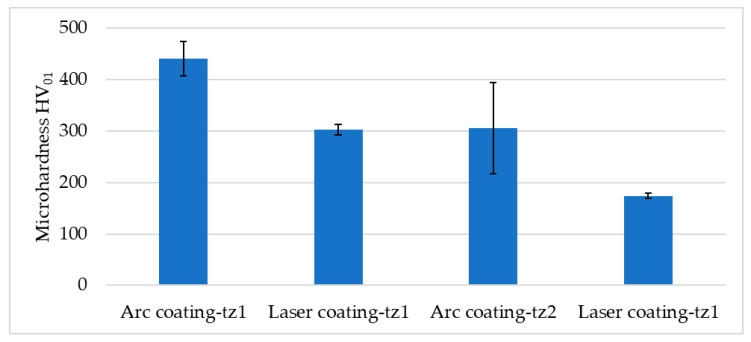
Results of the microhardness of the transition zones.

Additional hardness tests for the steel, along with the arc and laser coatings, included a study of the heat-treated zones formed during the coating application process. The study showed a decrease in the hardness values with distance from the heat-applied coating. For transition zone one, the values were about 120 HV_01_ higher for the arc coating compared to the laser coating. For transition zone two, the microhardness value was about 100 HV_01_ lower for the laser coating application process. Carbide separations for the laser coating were also observed, with a hardness value of 1588.9 HV_1_. The hardness results of the transition zones are presented in Figure 7.

**Figure 8:**
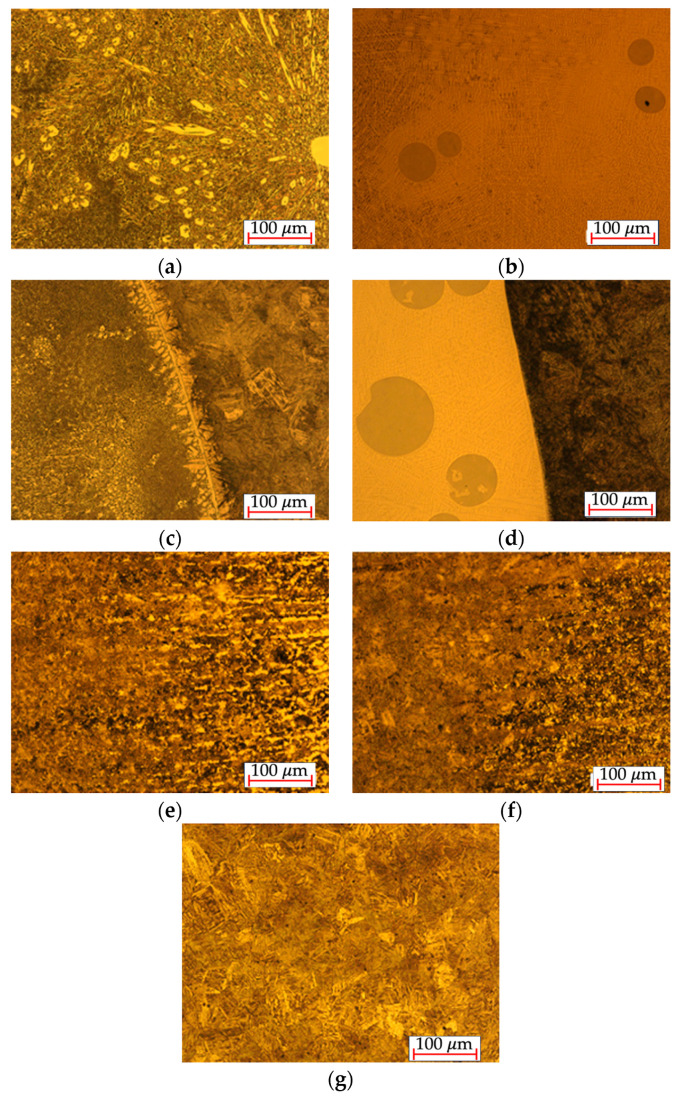
Microstructure of 28MnB5 steel: (**a**) arc coating, (**b**) laser coating, (**c**) transition zone 1 of the arc coating, (**d**) transition zone 1 of the laser coating, (**e**) transition zone 2 of the arc coating, (**f**) transition zone 2 of the laser coating, and (**g**) core material.

During the hardness tests, the microstructure view formed during coating application and for the native material was performed. The 28MnB5 steel is characterised by a martensitic structure. In the case of the laser coating, WC carbide separations (tungsten carbides) are noticeable, and the microstructure contains large amounts of chromium. In the case of the arc coatings, separations of chromium carbides are noticeable as the size of the heat-affected zone changes. With the distance from the heat-affected zone, the size of the grains formed in the material changes and the structure changes to fine-grained. Images of the microstructures are shown in Figure 8.

### 3.2. Results of Surface Roughness Tests

To determine the effect of the abrasive medium on the wear pattern, the roughness of the surface was examined after and before the tribological test. The aggregate results for both the laser and arc coatings are shown in Table 7 and Table 8.

For the laser coating, it was found that the increase in the values of the surface parameters R_z_, Rt, and R_a_ for the samples tested at a 10% moisture content was higher than for the samples tested at a 0% moisture concentration. The Ra value increased by about 223% for the samples tested on an abrasive mass with a 10% moisture content compared to an increase of approximately 106% for the samples with a 0% moisture content (the results were related to the results of the samples before the tribological test). The samples tested in the pH10 environment showed higher increases in the values of the roughness parameter than the samples tested in the pH7 environment. The R_z_ value increased by approximately 260% for the samples tested on the abrasive mass at a pH of 10 compared to an increase of approximately 110% for the samples at a pH of 7. The results indicate that the concentration of the substance (soil moisture), as well as the pH, influences the changes in the surface parameters after the tribological test. The samples tested at a higher moisture content and in an environment with a higher pH showed greater changes in the surface parameters, suggesting that these factors are crucial in evaluating the tribological behaviour of the materials.

Analysing the results of the tribological tests of the arc coating, it can be seen that the values of the surface parameters increased significantly for the samples tested at a 10% moisture content compared to the samples tested at a 0% moisture content. The R_z_ value increased by about 342% for the samples at a 10% moisture content, while for the samples at a 0% moisture content, the increase was only about 227%. The samples tested at a pH of 10 showed higher increases in roughness values than the samples tested at a pH of 7. The R_a_ value increased by about 407% for the pH10 samples compared to an increase of approximately 394% for the pH7 samples. The analysis of the results indicates that both the water content (soil moisture) and the pH had a significant effect on the changes in the surface parameters after the tribological test. The samples tested at a higher water content and in an environment with a higher pH level showed greater changes in the surface parameters, suggesting that these factors may be important in evaluating the tribological behaviour of the materials.

### 3.3. Tribological Experiment

When the results are compared for the different surface modification methods and environmental conditions, it can be observed that, compared to the base material, the arc coating significantly improved the tribological properties for the majority of combinations of abrasive medium conditions. The formula presented below was used to calculate the weight loss:

(2) $$Z_{v}=Mass\;before\;tribological\;test-mass\;after\;tribological\;test$$

**Figure 9:**
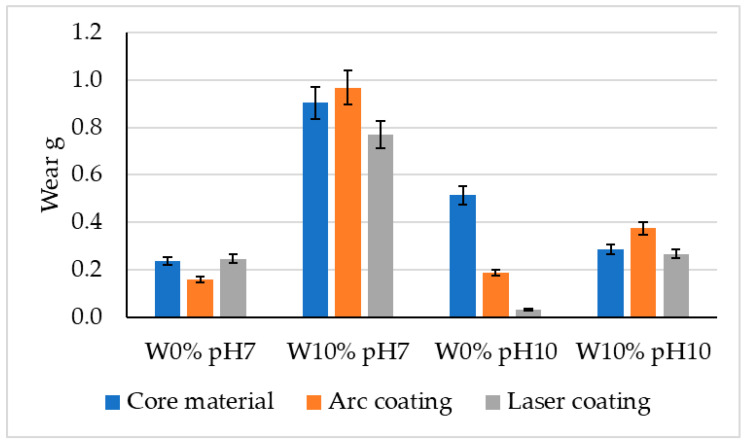
Results of the wear tests.

For the samples with a 10% moisture and a pH of 7, the mass loss value increased from 0.904 g to 0.968 g compared to the native material, indicating that the arc coating can be an effective method for improving the tribological properties under most conditions, except for elevated moisture conditions, as was also observed in samples with a 10% moisture and a pH of 10. The laser coating samples exhibited more varied effects compared to the arc coating samples. In some cases, there was an improvement in the tribological properties, while, in others, there was a deterioration. For the samples with a 0% water concentration and a pH of 10, the mass loss value decreased from 0.245 g to 0.031 g, suggesting a significant improvement in the tribological properties after the laser coating application. However, for the samples with a 10% moisture and a pH of 10, the mass loss value increased from 0.267 g to 0.374 g, indicating a deterioration in these properties. The cumulative research results are presented in Figure 9.

### 3.4. Power Consumption Results of the Test Stand

**Figure 10:**
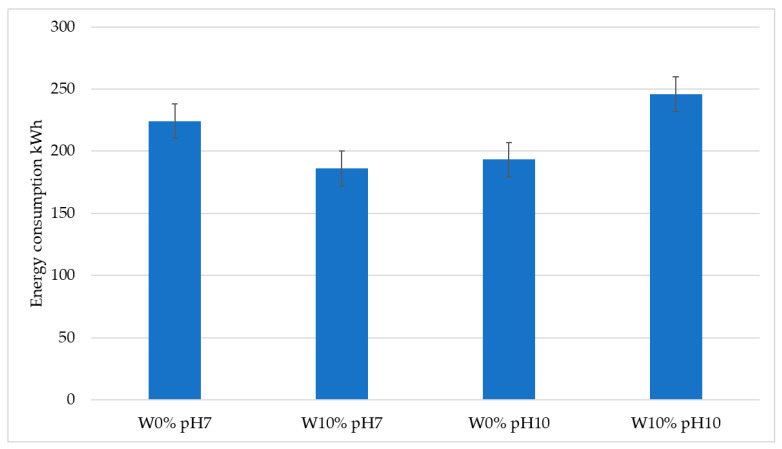
Results of the energy consumption tests during the wear tests, depending on the abrasive mass conditions.

The results of energy consumption were also recorded during the tribological tests. The results show the highest power consumption occurring for the 10% moisture content and the pH10 variant, and amounted to 245 kWh during the execution of a single tribological test. The lowest energy consumption was recorded for the 10% moisture and pH7 variant, which was lower than the highest value by about 60 kWh. The test results are presented in Figure 10.

**Figure 11:**
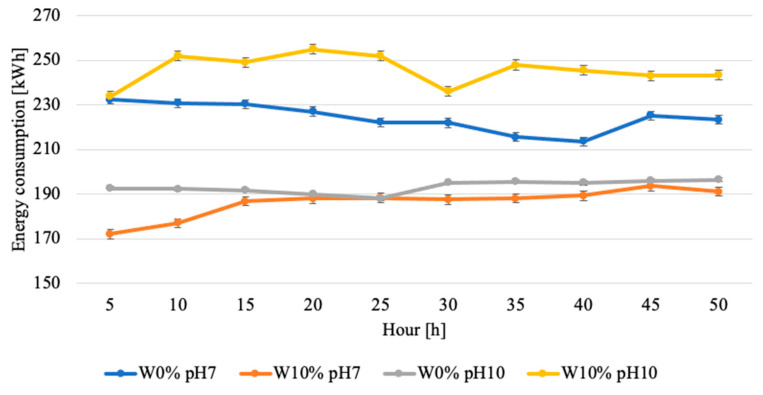
Results of the energy consumption tests during the wear tests, depending on abrasive mass conditions and the time of measurement.

Figure 11 also shows the distribution of the power consumption over the test. The power was measured every 5 h. For the sample with 10% moisture and a pH of 10, there is a noticeable decrease in the power consumption in the 30th hour of the test, which returns to a stabilised level in the following hours. For the sample with 0% moisture and a pH of 7, there is a slight decrease in the power consumption until the 40th hour, at which point there is an increase in the power support and then a decrease again. For the W10% and pH7 sample, there is a noticeable continuous increase in the power consumption with a stabilisation from hour 15 to hour 40 of the test. For the 0% moisture and pH10 sample, there is a slight decrease in the power consumption up to hour 25, while, from hour 30, the value of similar power consumption is almost constant.

Analysing the energy consumption in time, it can be seen that, for the highest moisture content and pH level, the energy consumption is higher due to overcoming the frictional resistance associated with compactness, and only after the initial stabilisation of the roughness does the power consumption stabilise. For the soil conditions of pH7 and W0%, the energy intake has a flat characteristic with a minimal decrease after the equilibrium roughness is achieved. For the W10% and pH7 soil conditions, the energy consumption characteristics are constant due to the constant surface area of the abrasive mass impact on the sample. For the W0% and pH10 test conditions, it is noticeable that the energy consumption is constant due to the change in the viscosity of the mass, which is caused by the addition of KOH to the abrasive medium, causing the dissolution of organic particles.

## 4. Discussion

**Figure 12:**
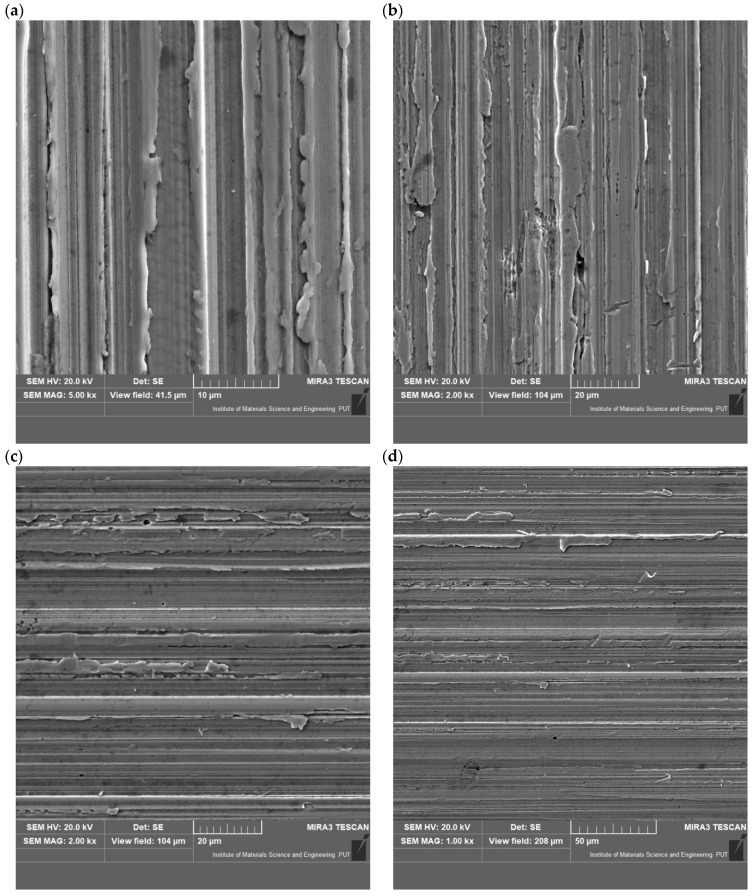
View of wear traces: (**a**) W10% pH7, (**b**) W10% pH10, (**c**) W0% pH7, and (**d**) W0% pH10.

This article presents research results related to the reduction in the abrasive wear of 28MnB5 steel after coating applications. Studies conducted under different abrasive mass conditions showed that, for a 10% moisture and a pH of 7, the loss of mass of elements was approximately four times greater than for conditions of 0% moisture and a pH of 7. The wear rate under these conditions was higher compared to the laser coating for 0% moisture and a pH of 10 by up to eight times; Jankaukas obtained similar results, who indicated that an increase in the WC content contributed to a ninefold increase in the wear resistance [36]. For heat-applied coatings, the greatest reduction in the wear occurred under conditions of 0% moisture and a pH of 10, which is seven times less than the wear of the base steel. Zeng’s research has shown that, with an increase in the pH level, the wear also increased three times compared to a neutral pH [37]. In the case of the condition with the highest type of wear (W10%, pH7), the arc coating exhibited greater wear than the laser coating and the native material. During Kuwika’s research, it was observed that the friction coefficient increased with the amount of water [38]. A similar relationship was observed for the W10% and pH10 sample in the research presented in this article. For 0% moisture and a pH of 7, the laser coating had a higher wear than the base material. Only in the case of 0% moisture and a pH of 10 did both the arc coating and the laser coating contribute to the reduction in the abrasive wear compared to the material without surface modification. Analysing the power consumption of the test bench during the experiments, it can be noticed that the lowest power consumption occurs for conditions with the highest mass wear of the tested elements (W10%, pH7), as also demonstrated in Airao’s research, indicating a double increase in energy consumption for wet samples compared to dry samples [39]. Furthermore, in the case of the increased wear of the base material, a lower power consumption is noticeable than in the variants with a low mass loss of the tested elements. Additionally, an assessment of the wear traces was carried out in order to analyse the mechanisms occurring on the coating surfaces (Figure 12). For the abrasive mass parameters of W10% and pH7, Figure 12a shows clear traces of the impact of the abrasive mass in the direction of the pressure on the sample. In addition, there is a microbrushing effect on the surface and corrosion processes on the effect of moisture. For this sample, the highest weight loss is noticeable. For parameters W10% and pH10 (Figure 12b), smaller traces of micro-cutting are observed, while there are higher values of microbrushing and delicate traces of micro-scratching. Visible traces of microbruising and scratching are observed for W0% and pH7 (Figure 12c). The effects of fatigue use typical of the microbrushing mechanism are noticeable. Micro-scratching and micro-scratching on the surface of the tested samples are also noticeable. The shape of the scratches formed indicates the direction of the pressure of the abrasive mass. In Figure 12d (W0% and pH10), the presence of cracks with a small depression, typical of the micro-scratching mechanism, is noticeable. A small proportion of micro-scratching can also be observed in the image. Traces of wear indicate the slight occurrence of the micro-scratching mechanism.

## 5. Conclusions

When the research results are taken into account, it can be concluded that the values of the selected parameters of the abrasive mass (the moisture content or pH) affect the increased power consumption and increased wear of the tested material (with and without coatings). The highest sample wear was observed for a 10% moisture and a pH of 7, regardless of the coating applied. The laser coating had the lowest wear in the environmental conditions of 0% moisture and a pH of 10, and was about 10 times lower than the wear of the arc coating tested in a 10% moisture and a pH of 7. In the case of 0% moisture and a pH of 7, the results of the sample weight loss are similar. This is related to the flow of the abrasive mass pushing on the tool and the properties of the abrasive mass (the compactness, which is related to the moisture content). In the future, the results of this study can be enriched with moisture gradation in order to learn more about the exact wear processes, as well as the analysis related to the pressure of the abrasive mass on the tools for different variants of the medium. The results presented can be used to develop a suitable shape for the working element and to reduce the power consumption through a structure designed for the type and properties of the soil in which the agricultural machinery with the tools operates.

## Figures and Tables

**Table 1:** Chemical composition of 28MnB5 steel in %.

Steel	C	Si	Mn	P	S	B	Cr	Mo	Ni	N	Al	Cu	Fe
28MnB5	0.29	0.3	1.2	0.14	0.03	0.28	0.03	0.01	0.02	0.58	0.27	0.04	balance

**Table 2:** Coating process parameters for the 28MnB5 steel.

Laser Surface Treatment Parameters					
gas	feeder rotation	laser power		speed	power density
Argon	1.2 rpm	1500 W		450 mm/min	120 W/mm^2^
MIG/MAG surface treatment parameters					
wire feeding	current	voltage	feed		
2.8 mm	115 A	16 V	4.5 mm/s		

**Table 3:** Chemical composition of the heat-applied coatings in %.

Coatings	C	Si	Mn	P	S	B	Cr	Mo	Ni
Arc coating	1.21	1.09	0.1	0.1	<0.01	<0.01	29.97	0.69	2
Laser coating	Stellite-6 + WC (70/30%)								

**Table 4:** Parameters of the abrasive mass.

moisture %	0	10
pH	7	10
Abrasive wear standard	ASTM G65 [11]	

**Table 5:** Indications of the abrasive medium.

	Moisture %	pH
W0pH7	0	7
W10pH7	10	7
W0pH10	0	10
W10pH10	10	10

**Table 6:** Work conditions of the test stand.

Research Conditions	Value
Rotational speed, rpm	100
Linear speed, km/h	13
Working time, h	50
Road, km	650
Immersion of the sample in the abrasive mass, mm	75
Intervals for replacing the abrasive mass, h	50

**Table 7:** Roughness of laser coatings before and after the tribological test in µm.

Laser Coating										
	Before Tribological Test					After Tribological Test				
	R_z_	R_t_	R_a_	S_z_	S_a_	R_z_	R_t_	R_a_	S_z_	S_a_
W0% pH7	14.26	76.94	2.64	217.93	11.92	20.40	18.30	3.78	72.76	2.12
W10% pH7	11.21	63.82	1.75	198.48	8.00	15.84	8.44	1.70	67.68	1.61
W0% pH10	11.45	78.15	1.83	148.55	7.03	44.83	46.81	9.67	99.85	8.47
W10% pH10	31.60	145.10	7.24	302.59	8.57	32.39	49.03	5.67	43.80	0.78

**Table 8:** Roughness of arc coatings before and after the tribological test in µm.

Arc Coating										
	Before Tribological Test					After Tribological Test				
	R_z_	R_t_	R_a_	S_z_	S_a_	R_z_	R_t_	R_a_	S_z_	S_a_
W0% pH7	2.39	1.55	0.32	8.36	0.23	61.42	123.89	9.37	152.83	4.13
W10% pH7	14.41	5.38	1.39	75.79	0.54	23.01	40.84	3.28	70.83	2.73
W0% pH10	16.59	23.92	3.15	91.04	2.04	77.17	161.98	15.97	183.83	11.64
W10% pH10	6.09	5.33	1.29	21.94	1.10	70.03	108.90	14.56	128.40	10.06

## Data Availability

The data presented in this study are available on request from the corresponding author.
